# Prognostic value of CC-chemokine receptor seven expression in patients with metastatic renal cell carcinoma treated with tyrosine kinase inhibitor

**DOI:** 10.1186/s12885-017-3065-3

**Published:** 2017-01-23

**Authors:** Yu Xia, Li Liu, Ying Xiong, Qi Bai, Jiajun Wang, Wei Xi, Yang Qu, Jiejie Xu, Jianming Guo

**Affiliations:** 1 Department of Urology, Zhongshan Hospital, Fudan University, 180 Fenglin Road, Shanghai, 200032 China; 2 Department of Biochemistry and Molecular Biology, School of Basic Medical Sciences, Fudan University, Mailbox 103, 138 Yixueyuan Road, Shanghai, 200032 China

**Keywords:** Metastatic renal cell carcinoma, CC-chemokine receptor 7, Overall survival, Progression free survival, Lymphatic invasion

## Abstract

**Background:**

CC-chemokine receptor seven (CCR7), a G-protein coupled receptor normally facilitating immune cells lymphatic homing, has recently been identified on several cancer cells in promoting invasion and lymphatic specific metastasis by mimicking normal leukocytes. As tyrosine kinase inhibitors for metastatic renal cell carcinoma (mRCC) mostly emphasized on vascular inhibition, whether the CCR7 expressing tumor cells with potential lymphatic invasion function could have an impact on mRCC patient’s drug response and survival, was unknown.

**Methods:**

In this study, in a clinical aspect, we retrospectively investigated the prognostic and predictive impact of tumoral CCR7 expression in 110 mRCC patients treated with sunitinib and sorafenib, and its correlation with pre- or post-administration lymphatic involvement. Immunohistochemistry on tissue microarrays were conducted for CCR7 expression evaluation.

**Results:**

Kaplan-Meier and univariate analyses suggested high tumoral CCR7 expression as an adverse prognosticator for mRCC patients’ overall survival (OS), which was further confirmed in the multivariate analyses (*P* = 0.002, *P* = 0.003 for bootstrap). This molecule could be combined with Heng’s risk model for better patient OS prediction. High tumoral CCR7 expression was also an independent dismal predictor for patients’ progression free survival (PFS) (*P* = 0.010, *P* = 0.013 for bootstrap), and correlated with poorer best drug response. Moreover, a possible correlation of CCR7 high expression and patients’ baseline and post-administration lymph node metastasis was found.

**Conclusions:**

High tumoral CCR7 expression correlated with potential lymphatic involvement and poor prognosis of mRCC patients treated with tyrosine kinase inhibitors. Further external validations and basic researches were needed to confirm these results.

**Electronic supplementary material:**

The online version of this article (doi:10.1186/s12885-017-3065-3) contains supplementary material, which is available to authorized users.

## Background

For patients with metastatic renal cell carcinoma (mRCC), therapeutic options have expanded significantly these years, since vascular endothelial growth factor (VEGF)-targeted tyrosine kinase inhibitor (TKI) drugs such as sunitinib and pazopanib have been established as first-line therapy [1]. Several clinical based prognostic models, for example the Heng’s risk criteria, have also achieved remarkable progress in mRCC patient survival prediction [2]. However, the objective response rates (ORRs) for most first-line TKI drugs were only around 30% [3], and the lack of validated molecular biomarkers impeded their personalized approach [4]. This was in contrast to many other tumor types, in which protein expression and mutation were used as basic accesses for drugs response and patient survival prediction [5, 6].

CC-chemokine receptor 7 (CCR7), a G-protein coupled receptor (GPCR) mostly expressed on immune cells, was initially regarded as an important regulator facilitating leukocytes homing to the lymphatic structures, where its two ligands CC-chemokine ligand 19 (CCL19) and CCL21 are constitutively expressed [7]. However, in recent years, aberrant high CCR7 expression has also been identified on several tumor types, linking to a potential invasive phenotype [8]. It has been suggested that CCR7 positive tumor cells could mimic the normal lymphocyte homing function and interact with lymph vessels, leading to subsequent lymph node specific metastasis [9].

Lymphatic and hematogenous disseminations were two regular metastasis pathways for malignancy. For mRCC, although the most common metastatic site was the lung, possibly via a hematogenous approach, local or distant lymph node involvement at diagnosis was not rare [10]. Patient receiving TKIs could also develop new lymph node metastasis during the treatment, leading to a progressive disease (PD). Several theories of TKI drug resistance emphasized an increase of tumor cell invasiveness after drug administration [11]. These processes mostly accompanied with tumor cell migration and matrix metalloproteinase-9 (MMP-9) mediated extracellular matrix degradation [12], which was similar to the CCR7 mediated lymph vessel intravasation process [8]. As VEGF targeted therapies mostly focused on blood vessels inhibition, whether they could enhance the possibility of mRCC metastasis through other pathways, such as CCR7 mediated lymph vessel invasion and therapy resistance, was not known.

Thus, here through immunohistochemistry (IHC), we retrospectively evaluated the CCR7 expression in 110 primary tumor specimens of mRCC patients treated with sunitinib and sorafenib. The result suggested a positive correlation of CCR7 expression with patient baseline lymph node metastasis and TKI drugs response. CCR7 high expression could predict a poorer overall survival (OS) and progression free survival (PFS) for mRCC patients after TKIs.

## Methods

### Patient selection

We initially screened a total of 138 mRCC patients treated with sunitinib or sorafenib between March 2005 and June 2014 at the Department of Urology, Zhongshan Hospital, Fudan University. The inclusion criteria were: pathologically proven RCC patient with metastatic lesion, treated with sunitinib or sorafenib at first without further second-line treatment, had enough Formalin Fixed Paraffin Embedded (FFPE) specimens, and had detailed laboratorial, imaging and survival information. Patients who had former malignant history, received metastasectomy or those with tumor necrosis area >80%, unavailable data were excluded. At last, 28 patients were excluded and 110 patients were selected for the study, in which three were excluded from PFS analysis for incomplete drug response information. This study was approved by the Clinical Research Ethics Committee of Zhongshan Hospital, Fudan University (Shanghai, China) (B2015-030).

Patients’ OS was defined as the time from therapy initiation to the time of death, or was censored at the last follow-up. PFS was calculated from the time of therapy initiation to the time of progression, according to the RECIST 1.1 criteria [13], or was censored at the last follow-up. All data were collected retrospectively from medical records and electronic databases using uniform database templates, and the last follow-up time was December 2015. According to the 2014 EAU guidelines [14] and 2012 ISUP consensus [15], two urologic pathologists (Yuan J. and Jun H.) independently reviewed all the H & E slides of patient samples and confirmed the RCC diagnosis and Fuhrman grade classification. Initial stage at diagnosis was reclassified based on the 2010 AJCC TNM classification [10]. Heng’s risk model was applied as previously reported [2].

### Tissue microarray and immunohistochemistry

Two representative tumor cores 3 mm in diameter from each sample were selected for tissue micro array (TMA) construction. Anti-CCR7 antibody (ab32527, Abcam, diluted 1/1000) and Dako EnVision Detection System were applied in the immunohistochemistry procedure [16]. Through western blot in RCC cell lines, the specificity of antibody was confirmed. Negative control was performed without applying primary antibody. Olympus CDD camera, Nikon eclipse Ti-s microscope (×200 magnification) and NIS-Elements F3.2 software were used to record the staining results. Using Image-Pro Plus version 6.0 software (Media Cybernetics Inc., USA), an integrated optical density (IOD) score could be calculated for each scan. Two urologists unaware of the patients’ clinical data evaluated these slides. Each person took three independent shots with the strongest staining for each core, and the IOD mean of each patient’s two cores (six scans) were calculated. Kappa value was calculated for evaluating inter-observer agreement.

### Statistical analysis

Univariate analysis was carried out to explore the prognostic and predictive value of continuous CCR7 IOD score. The smooth estimates of hazard ratio (HR) of IOD on patient survival were displayed using R software, “phenoTest” package [17]. For clinical usage, we dichotomized the IOD into high/low expression through minimum p value method using x-tile software [18], and because the p values obtained might be overestimated, they were corrected using the formula proposed by Altman and colleagues [19]. The smooth HR curves after dichotomizing were illustrated through R software, “smoothHR” package [20]. After this, *χ*2 test, Fisher’s exact method and Cochran-Mantel-Haenszel *χ*2 test were applied for assessing the relationship between CCR7 expression and patients’ clinicopathological parameters. Kaplan–Meier, univariate and subsequent multivariate analysis were performed, in which 1000 bootstrap resamples was used for reducing overfitting bias. Finally, time dependent receiver operating characteristic (ROC) analysis was done to analyze the adding prognostic value of CCR7 expression to the Heng’s risk model. GraphPad Prism 6 (GraphPad Software Inc., USA), SPSS 21.0 (SPSS Inc., USA), X-tile 3.6.1 (Robert L Camp, USA) and R software 3.1.2 (R Foundation for Statistical Computing, Austria) were used in these procedures. A two-sided *P*-*value* < 0.05 was regarded as statistically significant.

## Results

### CCR7 staining and cut off point choosing

CCR7 expression in the RCC sample was variable, and mostly on the membrane and cytoplasm of tumor cells (Additional file 1: Figure S1A and B). Its expression in peritumoral tissue was relatively low (Additional file 1: Figure S1C). Inter-observer agreement was acceptable according to the kappa value 0.745, thus the CCR7 IOD means from the two observers were again averaged as the final IOD, and the distributions were 73–584 for range; 238 ± 97 for mean and SD; 223 (173–287) for median and IQR.

In order to select an appropriate cut off value for clinical usage, first we performed a univariate analysis using the continuous CCR7 IOD score (Additional file 2: Table S1). Result suggested CCR7 as a significant adverse prognostic marker for mRCC patients’ OS and PFS (*P* < 0.001 for both). Smooth HR curve demonstrated the adding risk of 1 per IOD on patient survival (Additional file 1: Figure S1D and F). Then, through minimum p value method using log rank test, IOD = 215 was chosen as the cut off point (*P* < 0.001 for both), and the p values were still significant after being corrected (OS, *P* < 0.001, PFS, *P* = 0.001). The smooth HR curve also displayed significant and stable prognostic differences after dichotomizing (Additional file 1: Figure S1E and G).

### Patient baseline characteristics and its association with dichotomous CCR7 expression

The 110 patients’ baseline clinical characteristics were shown in Table 1. All patients were East Asian. The medium age at TKIs initiation was 59 years old (range 14–78). All patients have received radical, partial or cytoreductive nephrectomy. Results showed that the dichotomous CCR7 expression associated with TNM stage at initial diagnosis (*P* = 0.004), lymph node involvement (*P* = 0.031) and marginally with histology (*P* = 0.086) and number of metastatic sites (*P* = 0.070). It also correlated with patients’ best response of TKIs (*P* < 0.001).

**Table 1:** Clinical characteristics of patients according to tumoral CCR7 expression

Characteristics	Patients		Tumoral CCR7 expression		
	*n*	%	low	high	*P*-value
No. of patients	110	100	53	57	
Age, years, mean (SD)	57.5 (11.9)				
Men	79	71.8			
ECOG PS					0.198^c^
0	81	73.6	42	39	
1	29	26.4	11	18	
Prior nephrectomy					N/A
Yes	110	100.0			
No	0	0.0			
Histology					0.086^c^
Clear cell	88	80.0	46	42	
Non-clear cell type	22	20.0	7	15	
Papillary	15	13.6			
Chromophobe	2	1.8			
Collecting duct	2	1.8			
Unclassified	3	2.7			
Fuhrman grade (7 excluded)					0.147^d^
1	2	1.8	2	0	
2	53	48.2	28	25	
3	41	37.3	18	23	
4	7	6.4	5	2	
TNM stage at initial diagnosis					**0.004** ^c^
I-III	51	46.4	32	19	
IV	59	53.6	21	38	
Site of metastatic disease^a^					
Lung	82	74.5			
Liver	13	11.8			
Bone	18	16.4			
Lymph node	29	26.4			
Adrenal gland	8	7.3			
Brain and others	15	13.6			
Lymph node involvement^a^					**0.031** ^c^
No	81	73.6	44	37	
Yes	29	26.4	9	20	
No. of metastatic sites^a^					0.070^c^
1	76	69.1	41	35	
≥ 2	34	30.9	12	22	
Tyrosine kinase inhibitors					0.254^c^
Sunitinib	73	66.4	38	35	
Sorafenib	37	33.6	15	22	
Heng’s risk group					0.271^d^
Favorable	22	20	12	10	
Intermediate	60	54.5	30	30	
Poor	28	25.5	11	17	
Best response (3 not assessable)					**<0.001** ^d^
Partial response	27	25.2	18	9	
Stable disease for ≥3 months	57	53.3	30	27	
Progressive disease^b^	23	21.5	2	21	

Bold data means statistical significant (*P*<0.05)

*SD* standard deviation, *ECOG PS* Eastern Cooperative Oncology Group performance status

*P*-value < 0.05 was regarded as statistically significant

^a^At the time initializing tyrosine kinase inhibitors

^b^Including stable disease for <3 months

^c^χ^2^ test or Fisher’s exact test

^d^Cochran-Mantel-Haenszel *χ* ^2^ test

### Impact of baseline characteristics, including dichotomous CCR7 expression, on OS in mRCC patients receiving TKIs

**Fig. 1:**
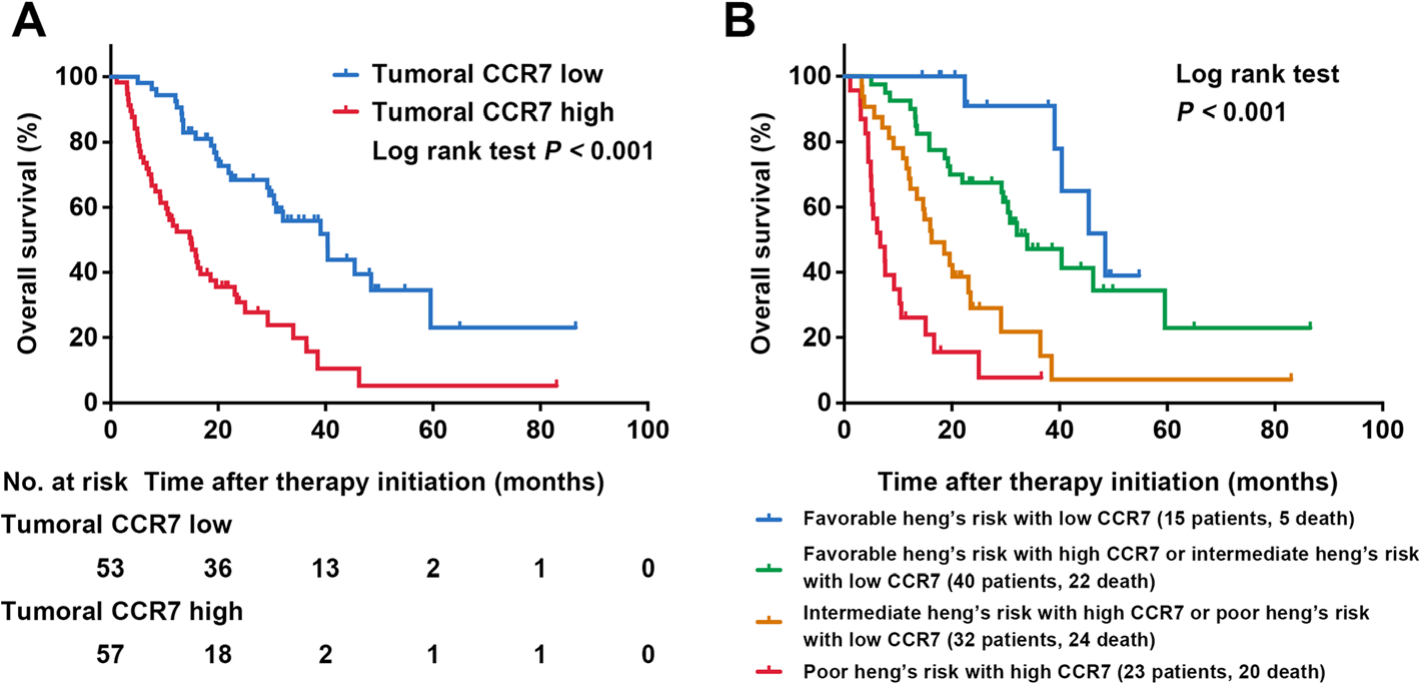
Impact of tumoral CCR7 expression on patients’ overall survival (OS). **a** OS according to tumoral CCR7 expression; **b** Heng’s risk model expanded with tumoral CCR7 expression

Within this cohort, 64.5% (71/110) patients died during the follow-up and the median OS was 23.5 months. Kaplan-Meier analysis revealed that patients with high CCR7 expression had a significantly poorer OS (*P* < 0.001 after correction) (Fig. 1a). Univariate analysis confirmed this significance (*P* < 0.001) (Additional file 2: Table S1). Multivariate Cox analysis was further performed and suggested high CCR7 expression as an independent adverse prognostic factor for mRCC patients’ OS prediction (HR 2.256, 95% CI 1.336–3.809, *P* = 0.002; *P* = 0.003 after 1000 bootstrap), together with tumor histology and Heng’s risk group (Table 2). Lung and lymph node involvement were excluded from the model for being potential confounding factors for number of metastatic sites.

**Table 2:** Multivariate analyses of characteristics associated with overall survival and progression free survival

Variables	OS (*n* = 110)					PFS (*n* = 107)				
	Original		Variables selected		Bootstrap^c^	Original		Variables selected		Bootstrap^c^
	HR (95%CI)	*P*-value^b^	HR (95%CI)	*P*-value^b^	*P*-value^b^	HR (95%CI)	*P*-value^b^	HR (95%CI)	*P*-value^b^	*P*-value^b^
Histology										
Non-clear cell vs clear cell	2.325 (1.293–4.181)	**0.005**	2.108 (1.191–3.732)	**0.010**	**0.011**	1.717 (1.008–2.926)	**0.047**	1.606 (0.952–2.710)	0.076	0.087
TNM stage at initial diagnosis										
IV vs I–III	0.990 (0.565–1.736)	0.972				0.988 (0.599–1.631)	0.963			
No. of metastatic sites^a^										
≥ 2 vs 1	1.465 (0.867–2.477)	0.154				1.946 (1.210–3.128)	**0.006**	1.817 (1.138–2.902)	**0.012**	**0.026**
Tyrosine kinase inhibitors										
Sorafenib vs Sunitinib	1.431 (0.861–2.381)	0.167				1.360 (0.858–2.154)	0.191			
Heng’s risk group		**<0.001**		**<0.001**	**0.005**		**0.036**		**0.011**	**0.035**
Favorable	reference		reference			reference		reference		
Intermediate	1.955 (0.895–4.268)		2.023 (0.964–4.244)			1.212 (0.634–2.313)		1.297 (0.701–2.398)		
Poor	5.654 (2.267–14.103)		6.760 (2.932–15.587)			2.432 (1.095–5.398)		2.771 (1.326–5.788)		
Tumoral CCR7										
High vs Low	2.242 (1.288–3.901)	**0.004**	2.256 (1.336–3.809)	**0.002**	**0.003**	1.782 (1.105–2.875)	**0.018**	1.835 (1.156–2.912)	**0.010**	**0.013**

Bold data means statistical significant (*P*<0.05)

*HR* Hazard Ratio, *CI* confidence interval, *OS* overall survival, *PFS* progression free survival

*P*-value <0.05 was regarded as statistically significant

^a^At the time initializing tyrosine kinase inhibitors

^b^Data obtained from the Cox proportional hazards model

^c^Bootstrapping with 1000 resamples were used

**Fig. 2:**
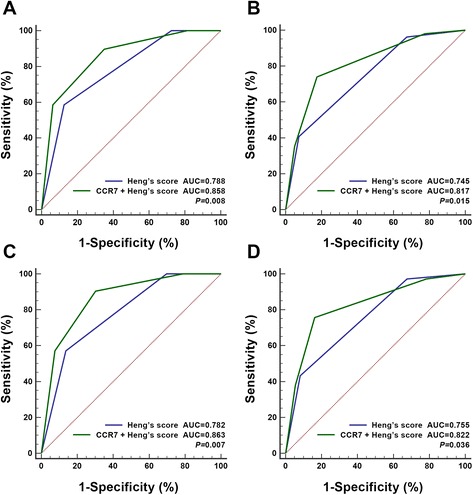
ROC analysis of Heng’s risk model alone and expanded with CCR7 expression on patients’ OS. **a** all patients at 12 months; **b** all patients at 24 months; **c** pathologic clear cell type at 12 months; **d** pathologic clear cell type at 24 months

Stratified analysis were further performed, and we found that CCR7 expression could discriminate most patient groups’ overall survival except those in the non-clear cell type or Heng’s risk favorable/poor groups (Additional file 3: Table S2). But after incorporating the high/low CCR7 expression directly into Heng’s risk to form a new model, the OS between different groups displayed vigorously discriminative consequences (*P* < 0.001) (Fig. 1b). Furthermore, ROC analysis was carried out at the time of 12 and 24-month follow-up, and the new model showed better prognostic power than using Heng’s risk model alone in both the ccRCC and all patient groups (Fig. 2). Incorporating CCR7 IOD score as a continuous variable also displayed similar results (Additional file 4: Figure S2).

### Impact of baseline characteristics, including dichotomous CCR7 expression, on PFS in mRCC patients receiving TKIs

**Fig. 3:**
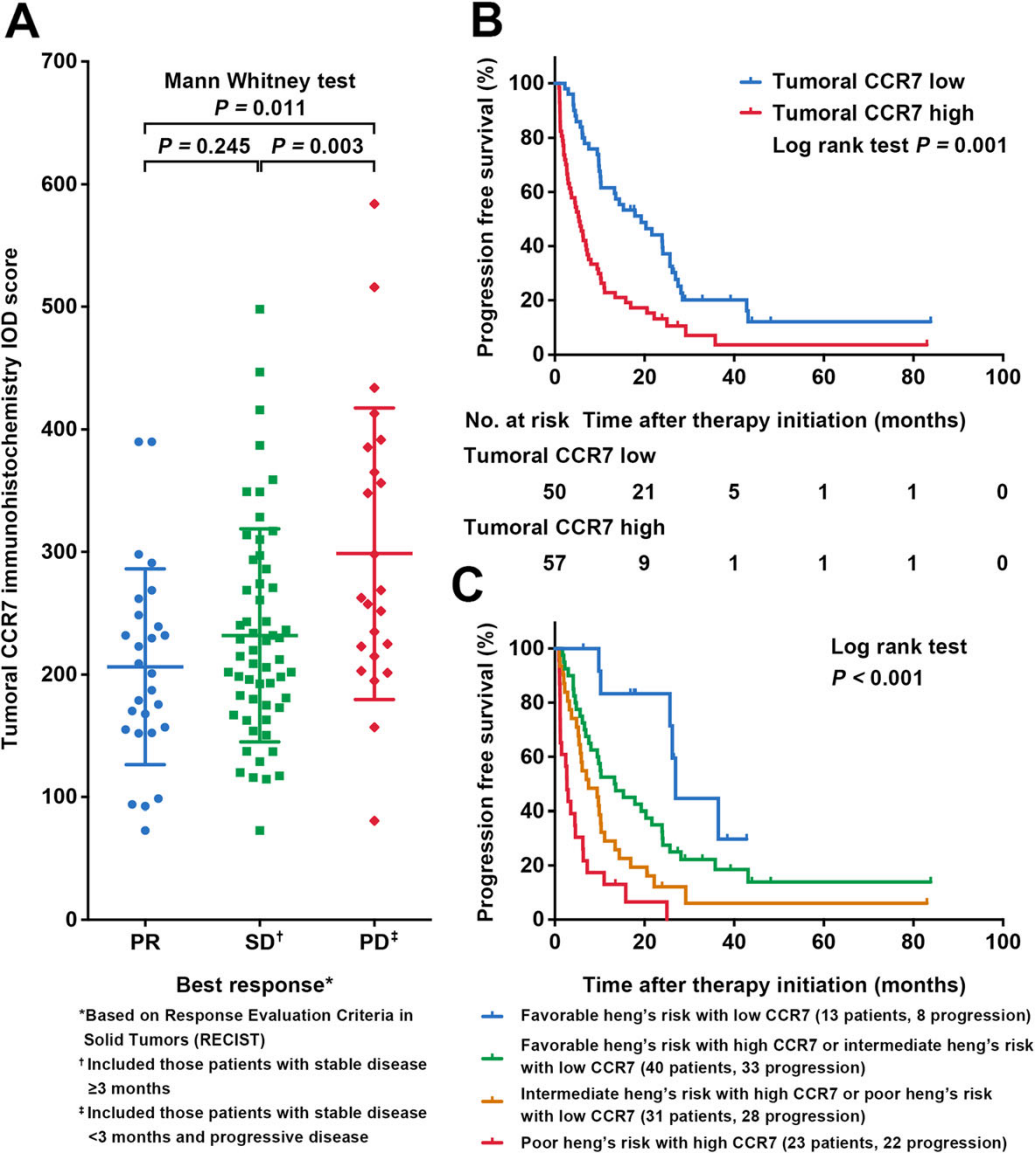
Impact of tumoral CCR7 expression on patients’ best drug response for tyrosine kinase inhibitors and PFS. **a** Patients’ best drug response according to tumoral CCR7 expression; **b** PFS according to tumoral CCR7 expression; **c** Heng’s risk model expanded with tumoral CCR7 expression

During the follow-up period, 85.0% (91/107) patients have developed disease progression. The median PFS was 9.8 months. Patients’ best response and its correlation with CCR7 were shown in Table 1. Figure 3a revealed that RCC in the PD group displayed a significantly higher CCR7 expression compared to partial response (PR) and stable disease (SD) groups. Kaplan-Meier analysis suggested an adverse predictive effect of high CCR7 expression in patients receiving TKIs (*P* = 0.001 after correction) (Fig. 3b) and was also confirmed in a multivariate model (HR 1.835, 95% CI 1.156–2.912, *P* = 0.010; *P* = 0.013 after 1000 bootstrap) (Table 2). After incorporating CCR7 into the Heng’s model, patients in the new model displayed significant PFS divergence between different groups (*P* < 0.001) (Fig. 3c). Since the Heng’s risk criteria was initially designed for OS prediction, further ROC comparison was not performed.

### CCR7 expression and its correlation with lymph node involvement

**Fig. 4:**
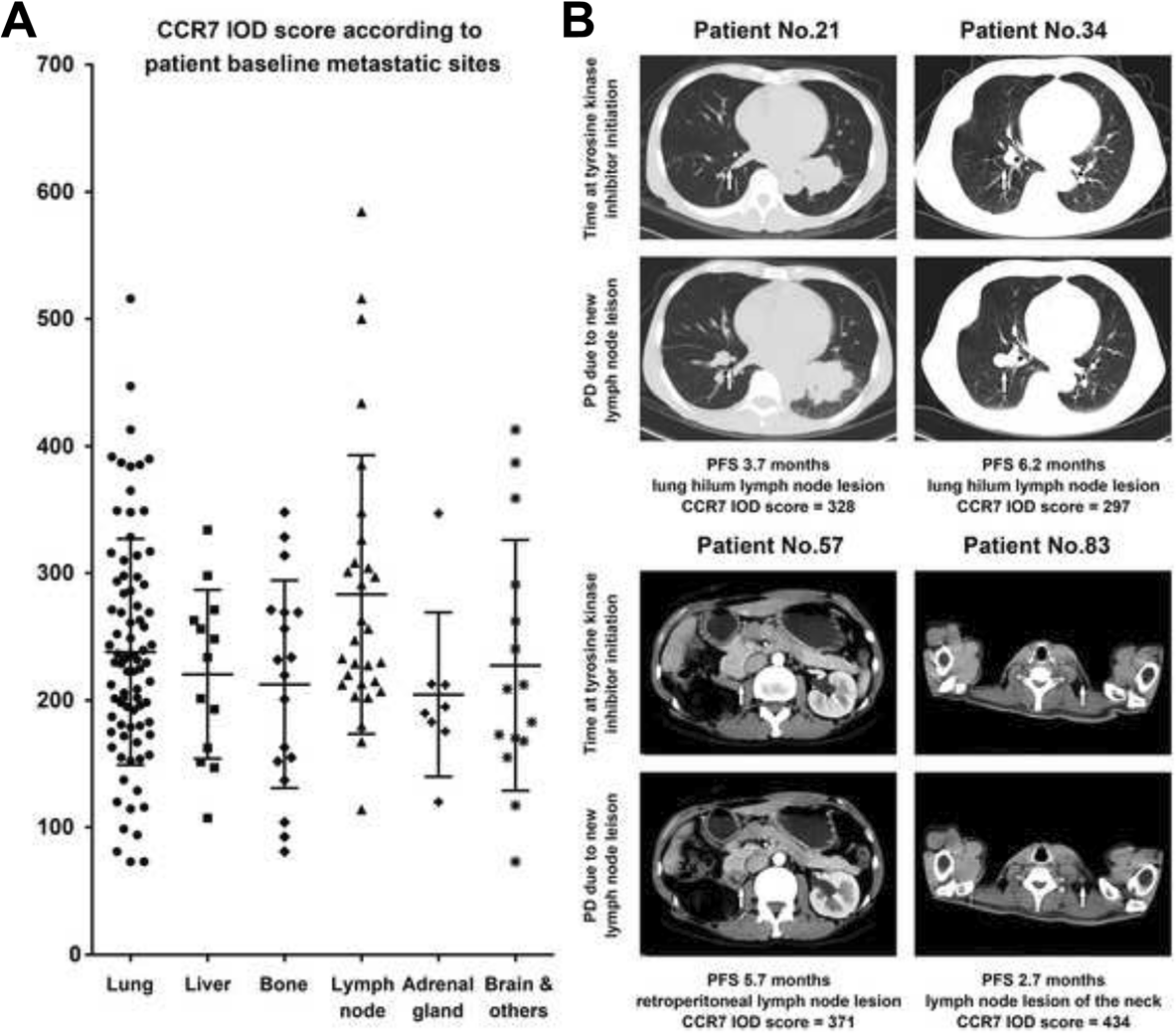
Correlation of tumoral CCR7 expression and patients’ baseline and post-administration lymphatic involvements. **a** Tumoral CCR7 expression according to different patient baseline metastatic sites; **b** Four mRCC patients who have experienced disease progression due to new lymphatic lesions development after tyrosine kinase inhibitors, all with high CCR7 expression. White arrow: the area where new lymph node lesions developed during administration

In Fig. 4a, the CCR7 IOD score of mRCC patients with different baseline metastatic sites were plotted, and revealed a potential higher expression of CCR7 in patients with baseline lymph node metastasis, in accordance with the *χ*2 test in Table 1, though the Kruskal-Wallis test did not meet statistical significance (*P* = 0.083). For exploring the possible impact of CCR7 on lymphatic invasion during the drug treatment period, we found that four patients within this cohort have developed disease progression due to new lymph node lesions development, and all their tumor samples displayed CCR7 high expression (Fig. 4b).

## Discussion

CCR7 was naturally a homeostatic chemokine receptor expressed on various subtypes of immune cells encompassing T cells, B cells, natural killer cells and dendritic cells, enabling them to circulate through the CCL-19/21 positive lymphatic highways [7]. Its expression on cancer cells was first recognized on hematogenous malignancies, in which the connection between high CCR7 expression and lymphoid organ involvement was discovered [21]. Subsequently, numerous laboratory studies confirmed this pro-invasion, mostly pro-lymph vessel metastatic function in various cancer types, including breast cancer, melanoma, non-small cell lung cancer, prostate cancer, head-and-neck cancer and gastrointestinal cancer [9]. Here, our results suggested that CCR7 also expressed on several RCC specimens and associated with various patient baseline characteristics (Table 1, Additional file 1: Figure S1).

The clinical prognostic value of CCR7 has been studied in several other cancer types. In a meta-analysis including 1697 gastric cancer patients, high CCR7 correlated with a worse 5-year overall survival rate [22]. Studies in melanoma and colorectal cancer also displayed similar results [23, 24]. In this study, we have found that high CCR7 staining intensity could be used as an adverse prognosticator for mRCC patients’ overall survival. Moreover, a new model integrating CCR7 expression into Heng’s risk criteria performed better than using Heng’s risk alone, and the adding prognostic value mostly came from the intermediate patient groups (Additional file 3: Table S2, Fig. 2a and b). This indicated that a substantial number of patients might switch between risk groups with a consequence for choice of treatment strategy after CCR7 status was considered. ROC analysis for the ccRCC patient group were also carried out, considering the poor performance of CCR7 in non-ccRCC patients (Fig. 2c and d).

For PFS analysis, to our knowledge, this study was the first to report an association between tumoral CCR7 expression and PFS in mRCC patients following TKIs. As the most widely used systemic therapy at present, TKI drugs only reached ORRs for about 30% for mRCC patients, and the theories of drug resistance was complicated [25]. It is increasingly evident that in some tumors, in which angiogenesis is thwarted genetically or pharmacologically, cancer cells could adapt by migrating more aggressively into normal tissue, based on several pre-existing invasion programmes such as epithelial-mesenchymal transition (EMT) and MMP2/MMP9 secretion [26, 27], or by switching on several distinctive programmes which were currently unknown [28]. Since the CCR7 mediated lymphatic specific migration and metastasis was also based on several above mentioned programmes [8, 29], and as a molecule which could give tumor itself survival signal besides [30], we hypothesized that CCR7 positive RCC cells might have a potential to migrate into the adjacent lymphatic tissue for survival after TKI drugs administration, subsequently leading to resistance and disease progression. As a result, our study did identify an independent significant adverse predictive effect of high CCR7 expression on mRCC patients’ PFS (Fig. 3b) and its association with poorer best drug response (Table 1, Fig. 3a). Patients’ baseline lymph node involvement status was also correlated (Table 1, Fig. 4a) and four patients who developed PD due to new lymph node metastasis all represented high CCR7 staining for their tumor tissues. All these results above indicated an impact of tumoral CCR7 expression on patients’ PFS and lymphatic involvement status, making this molecule a potential predictor for mRCC patients receiving TKIs.

The major limitations of this pilot study were its retrospective design and relatively small sample size, with patients from a single center and same ethnic background. Although central patient data review and bootstrap validation were performed for minimizing inter-observer and over-fitting bias, further external prospective validations were required, and related basic researches were needed for verifying our hypothesis. Samples from the metastatic site after TKI therapies might be excellent candidates for in-depth investigation. Besides, although we took two cores and six scans from each patient’s sample, intratumoral heterogeneity still might confound the results. Patients in this study received sunitinib or sorafenib as first-line therapy because other agents were not available in China at that time, and other VEGF-based TKI drugs such as pazopanib, axitinib and cabozantinib should also be taken into further consideration.

## Conclusions

Our study indicated that high tumoral CCR7 expression correlated with potential lymphatic involvement and poor prognosis in mRCC patients treated with TKIs. This biomarker could also be combined with the Heng’s risk model for better risk stratification.

## Additional files

Additional file 1:
**Figure S1.** Representative photographs of CCR7 immunostaining and cut-off point choosing. (A) Tumoral CCR7 low expression; (B) Tumoral CCR7 high expression; (C) Peritumoral CCR7 expression. Original magnification × 200; (D), (F) Smooth estimates of HR (+1 IOD) showed a higher risk of death and progression for patients with stronger tumoral CCR7 staining; (E), (G) Smooth estimates of HR (using IOD = 215 as a reference) showed a significant and stable prognostic difference between patients with high/low tumoral CCR7 staining. Dashed lines: 95% confidence bands. (TIF 5423 kb)
Additional file 2:
**Table S1.**Univariate analyses of characteristics associated with overall survival and progression free survival. (DOCX 69 kb)
Additional file 3:
**Table S2.**Hazard ratios for OS and PFS based on CCR7 in different subgroups (High vs Low). (DOCX 63 kb)
Additional file 4:
**Figure S2.**ROC analysis of Heng’s risk model alone and expanded with CCR7 continuous IOD score on patients’ OS. (A) all patients at 12 months; (B) all patients at 24 months; (C) pathologic clear cell type at 12 months; (D) pathologic clear cell type at 24 months. (TIF 1371 kb)

## Abbreviations

CCR7: CC-chemokine receptor 7
ccRCC: Clear-cell renal cell carcinoma
HR: Hazard ratio
IHC: Immunohistochemistry
IOD: Integrated optical density
mRCC: Metastatic renal cell carcinoma
ORR: Objective response rate
OS: Overall survival
PD: Progressive disease
PFS: Progression free survival
RCC: Renal cell carcinoma
ROC: Receiver operating characteristic
TKI: Tyrosine kinase inhibitor
