# Supplementary material for: Prognostic value of CC-chemokine receptor seven expression in patients with metastatic renal cell carcinoma treated with tyrosine kinase inhibitor
Source: BMC Cancer. 2017 Jan 23;17:70. doi: 10.1186/s12885-017-3065-3 (PMC5259971; doi:10.1186/s12885-017-3065-3)
Supplement: Additional file 2: — Table S1.Univariate analyses of characteristics associated with overall survival and progression free survival. (DOCX 69 kb) [file 12885_2017_3065_MOESM2_ESM.docx]

| **Table S1: Univariate analyses of characteristics associated with overall survival and progression free survival** | | | | | | | |
| --- | --- | --- | --- | --- | --- | --- | --- |
| **Variables** | **OS (n=110)** | | |  | **PFS (n=107)** | | |
|  | **Hazard Ratio** | **95%CI** | **P-value**† |  | **Hazard Ratio** | **95%CI** | **P-value**† |
| Age, years |  |  |  |  |  |  |  |
| Per 1 year increase | 0.985 | 0.968-1.004 | 0.118 |  | 0.989 | 0.972-1.006 | 0.195 |
| Gender |  |  |  |  |  |  |  |
| Male *vs* Female | 0.966 | 0.582-1.605 | 0.894 |  | 1.311 | 0.813-2.115 | 0.266 |
| Histology |  |  |  |  |  |  |  |
| Non-clear cell *vs* clear cell | 2.102 | 1.220-3.621 | **0.007** |  | 1.601 | 0.979-2.619 | 0.061 |
| Fuhrman grade (7 excluded) |  |  |  |  |  |  |  |
| 3+4 *vs* 1+2 | 1.424 | 0.870-2.333 | 0.160 |  | 1.117 | 0.727-1.716 | 0.613 |
| TNM stage at initial diagnosis |  |  |  |  |  |  |  |
| IV *vs* I-III | 2.081 | 1.284-3.374 | **0.003** |  | 1.768 | 1.162-2.691 | **0.008** |
| Lung involvement* |  |  |  |  |  |  |  |
| Yes *vs* No | 2.076 | 1.113-3.873 | **0.022** |  | 1.838 | 1.081-3.126 | **0.025** |
| Lymph node involvement* |  |  |  |  |  |  |  |
| Yes *vs* No | 1.905 | 1.144-3.173 | **0.013** |  | 2.449 | 1.533-3.914 | **<0.001** |
| No. of metastatic sites* |  |  |  |  |  |  |  |
| ≥2 *vs* 1 | 1.853 | 1.140-3.011 | **0.014** |  | 2.226 | 1.438-3.445 | **<0.001** |
| Tyrosine kinase inhibitors |  |  |  |  |  |  |  |
| Sorafenib *vs* Sunitinib | 1.576 | 0.994-2.535 | 0.053 |  | 1.360 | 0.882-2.097 | 0.164 |
| Heng’s risk group |  |  | **<0.001** |  |  |  | **<0.001** |
| Favorable | reference | - |  |  | reference | - |  |
| Intermediate | 2.219 | 1.066-4.620 |  |  | 1.500 | 0.827-2.721 |  |
| Poor | 8.193 | 3.658-18.350 |  |  | 4.206 | 2.152-8.220 |  |
| Tumoral CCR7 |  |  |  |  |  |  |  |
| IOD per 1 point increase | 1.005 | 1.003-1.007 | **<0.001** |  | 1.005 | 1.003-1.007 | **<0.001** |
| High *vs* Low | 3.153 | 1.919-5.180 | **<0.001** |  | 2.377 | 1.557-3.630 | **<0.001** |
| *At the time initializing tyrosine kinase inhibitors; CI=confidence interval; OS= overall survival; PFS= progression free survival; †Data obtained from the Cox proportional hazards model, P**-**value <0.05 was regarded as statistically significant | | | | | | | |
