# Supplementary material for: Prognostic value of CC-chemokine receptor seven expression in patients with metastatic renal cell carcinoma treated with tyrosine kinase inhibitor
Source: BMC Cancer. 2017 Jan 23;17:70. doi: 10.1186/s12885-017-3065-3 (PMC5259971; doi:10.1186/s12885-017-3065-3)
Supplement: Additional file 3: — Table S2.Hazard ratios for OS and PFS based on CCR7 in different subgroups (High vs Low). (DOCX 63 kb) [file 12885_2017_3065_MOESM3_ESM.docx]

| **Table S2: Hazard ratios for OS and PFS based on CCR7 in different subgroups (High *vs* Low)** | | | | | | | |
| --- | --- | --- | --- | --- | --- | --- | --- |
| **Patient subgroups** | **OS (n=110)** | | |  | **PFS (n=107)** | | |
|  | **Hazard Ratio** | **95%CI** | **P-value**† |  | **Hazard Ratio** | **95%CI** | **P-value**† |
| Histology |  |  |  |  |  |  |  |
| Clear cell | 3.187 | 1.813-5.602 | **<0.001** |  | 2.303 | 1.426-3.719 | **0.001** |
| Non-clear cell | 1.881 | 0.656-5.391 | 0.240 |  | 2.391 | 0.909-6.292 | 0.077 |
| TNM stage at initial diagnosis |  |  |  |  |  |  |  |
| I-III | 4.230 | 1.854-9.652 | **0.001** |  | 3.207 | 1.643-6.259 | **0.001** |
| IV | 2.316 | 1.213-4.421 | **0.011** |  | 1.687 | 0.958-2.972 | 0.070 |
| No. of metastatic sites* |  |  |  |  |  |  |  |
| 1 | 2.758 | 1.506-5.051 | **0.001** |  | 2.221 | 1.317-3.744 | **0.003** |
| ≥2 | 3.355 | 1.348-8.354 | **0.009** |  | 2.499 | 1.151-5.425 | **0.021** |
| Lymph node involvement* |  |  |  |  |  |  |  |
| No | 2.884 | 1.605-5.183 | **<0.001** |  | 2.317 | 1.405-3.821 | **0.001** |
| Yes | 3.662 | 1.364-9.833 | **0.010** |  | 2.430 | 0.988-5.876 | 0.057 |
| Tyrosine kinase inhibitors |  |  |  |  |  |  |  |
| Sunitinib | 3.323 | 1.784-6.192 | **<0.001** |  | 2.276 | 1.356-3.819 | **0.002** |
| Sorafenib | 4.327 | 1.511-12.387 | **0.006** |  | 2.674 | 1.194-5.989 | **0.017** |
| Heng’s risk group |  |  |  |  |  |  |  |
| Favorable | 3.676 | 0.886-15.244 | 0.073 |  | 2.345 | 0.767-7.171 | 0.135 |
| Intermediate | 2.363 | 1.228-4.546 | **0.010** |  | 1.794 | 1.025-3.139 | **0.041** |
| Poor | 2.322 | 0.779-6.917 | 0.130 |  | 1.596 | 0.540-4.713 | 0.397 |
| *At the time initializing tyrosine kinase inhibitors; CI=confidence interval; OS= overall survival; PFS= progression free survival; †Data obtained from the Cox proportional hazards model, P**-**value <0.05 was regarded as statistically significant | | | | | | | |
