# Supplementary figures and images for: Prognostic value of CC-chemokine receptor seven expression in patients with metastatic renal cell carcinoma treated with tyrosine kinase inhibitor
Source: BMC Cancer. 2017 Jan 23;17:70. doi: 10.1186/s12885-017-3065-3 (PMC5259971; doi:10.1186/s12885-017-3065-3)

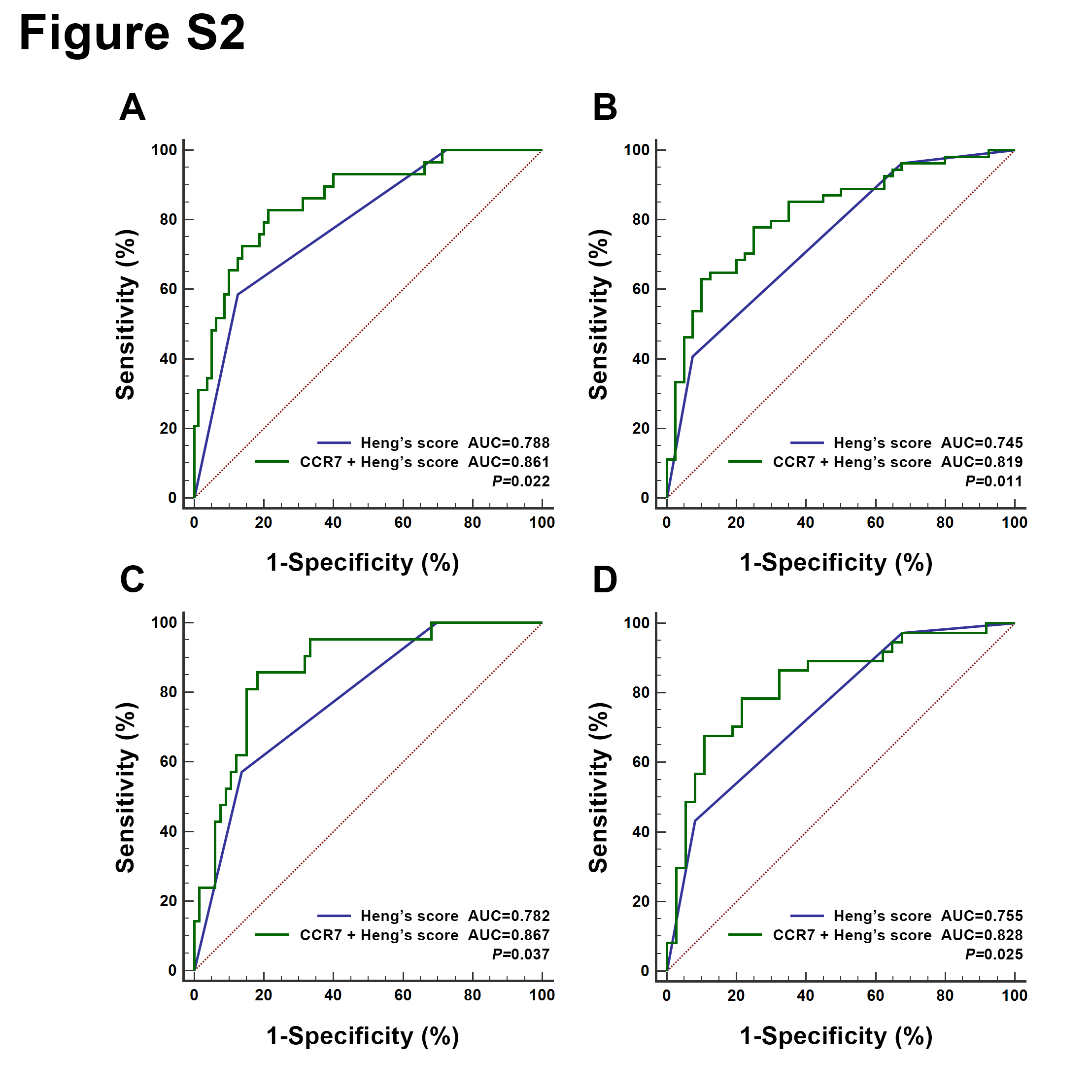

Supplement: Additional file 4: — Figure S2.ROC analysis of Heng’s risk model alone and expanded with CCR7 continuous IOD score on patients’ OS. (A) all patients at 12 months; (B) all patients at 24 months; (C) pathologic clear cell type at 12 months; (D) pathologic clear cell type at 24 months. (TIF 1371 kb) [file 12885_2017_3065_MOESM4_ESM.tif]
